# May Young Elite Cyclists Have Less Efficient Bone Metabolism?

**DOI:** 10.3390/nu11051178

**Published:** 2019-05-26

**Authors:** Marta Rapún-López, Hugo Olmedillas, Alejandro Gonzalez-Agüero, Alba Gomez-Cabello, Francisco Pradas de la Fuente, Luis A. Moreno, José A. Casajús, Germán Vicente-Rodríguez

**Affiliations:** 1 Departamento de Expresión Musical, Plástica y Corporal, Facultad de Ciencias de la Salud y del Deporte, Universidad de Zaragoza, Huesca, C/Ronda Misericordia, 5, 22001 Huesca, Spain; mrapun@unizar.es (M.R.-L.); franprad@unizar.es (F.P.d.l.F.); 2 Department of Functional Biology, Universidad de Oviedo, Campus del Cristo B. Julián Clavería s/n, 33006 Asturias, Spain; olmedillashugo@uniovi.es; 3 GENUD (Growth, Exercise, NUtrition and Development) Research Group, Universidad de Zaragoza, 50009 Zaragoza, Spain; alexgonz@unizar.es (A.G.-A.); agomez@unizar.es (A.G.-C.); lmoreno@unizar.es (L.A.M.); joseant@unizar.es (J.A.C.); 4 Department of Physiatry and Nursing, Faculty of Health and Sport Sciences (FCSD), University of Zaragoza, Ronda Misericordia 5, 22001 Huesca, Spain; 5 Centro de Investigación Biomédica en Red de Fisiopatología de la Obesidad y Nutrición (CIBERObn), 28029 Madrid, Spain; 6 Instituto Agroalimentario de Aragón (IA2), 50013 Zaragoza, Spain; 7 Centro Universitario de la Defensa, 50090 Zaragoza, Spain; 8 Department of Physiatry and Nursing, Faculty of Health Sciences, University of Zaragoza, Calle Domingo Miral, s/n, 50009 Zaragoza, Spain

**Keywords:** cyclists, adolescence, bone turnover, osteocalcin, vitamin D

## Abstract

The purpose of this work was to describe changes in metabolic activity in the bones of young male competitive cyclists (CYC) as compared with age-matched controls (CON) over a one-year period of study. Eight adolescent male cyclists aged between fourteen and twenty, and eight age-matched controls participated in this longitudinal study. Serum osteocalcin (OC), amino-terminal propeptide of type I procollagen (PINP), beta-isomerized C-telopeptides (β-CTx) and plasma 25 hydroxyvitamin D [25(OH)D], were investigated by an electrogenerated chemiluminescence immunoassay. Analysis of variance revealed no significant differences in formation and resorption markers between cyclists and controls. Within the groups, both CYC and CON showed decreased OC at −30% and −24%, respectively, and PINP where the figures were −28% and −30% respectively (all *p* < 0.05). However, only the CYC group showed a decrease in [25(OH)D], lower by 11% (*p* < 0.05). The similarity in the concentrations of markers in cyclists and controls seems to indicate that cycling does not modify the process of bone remodeling. The decrease in vitamin D in cyclists might be detrimental to their future bone health.

## 1. Introduction

Osteoporosis is a serious skeletal disease which continues to grow in our society, characterized by low bone mineral density (BMD) and microarchitectural deterioration of bone tissue, affected by the peak of bone mass obtained before 20 years of age [1]. People affected by osteoporosis present bone fragility and fracture risk, with a consequent deterioration in quality of life [2], and increased mortality [3]. Therefore, the prevention of the development of the disease is crucial and it should start during adolescence [4].

Several studies suggested that maximizing bone mineral acquisition during growth might reduce the risk of osteoporosis in later life [5,6,7,8]. Bone health and positive metabolic balance are influenced by genetic and environmental factors, including physical activity. Exercise during adolescence may contribute to the prevention of osteoporosis, although it needs to be a weight-bearing activity to be osteogenic [5,9]. In this sense, cycling may adversely affect bone mass during adolescence [10], and several studies have diagnosed osteopenia and osteoporosis in professional and master cyclists [11,12,13].

Bone development depends mainly on bone turnover, which includes bone formation and resorption [14], and may be estimated by different biochemical markers, osteocalcin (OC) and amino-terminal propeptide of type I procollagen (PINP) as markers of bone formation, and serum or urine beta-isomerized C-telopeptides (β-CTx) as a marker of bone resorption. The bone metabolism markers have been widely used to determinate the variations in the bone remodeling process as a consequence of physical activity [15,16,17], and indicate modeling and remodeling of bone tissue during pubertal growth and maturation [18]. 

Higher values of bone formation and resorption markers have been found in adolescent athletes [10,19], although similar results among athletes and controls have also been determined [20]. Additionally, it is important to know the vitamin D status because of the influence that this nutrient has on bone mass and bone loss [21]. Positive and direct associations between plasma 25 hydroxyvitamin D [25(OH)D] (active form of vitamin D) and bone mineral content (BMC) were previously described [21,22,23,24], however, there is a scarce amount of information on young cyclists, a population that may be at a higher risk due to their non-osteogenic sport participation. 

Despite the importance of assessing bone metabolism in adolescents, there has been little research on the association between biochemical markers and sport participation, and most are cross-sectional studies. However, longitudinal studies may help us to understand how sport can influence bone metabolism, and how this is translated to bone development, which is crucial information to cyclists who seem to be at a higher risk of not achieving optimal peak bone mass [25,26]. 

The purpose of our longitudinal study was to measure the effect of cycling on bone metabolism in adolescent cyclists and to compare it to active age-matched peers.

## 2. Materials and Methods

### 2.1. Study Design

A one-year longitudinal design was used, with repeated measurements performed at the beginning and at the end of the season, in November and in October.

### 2.2. Ethics Statement

Written informed consent was obtained from parents and adolescents. The study was performed following the ethical guidelines of the Declaration of Helsinki 1961 (revision of Fortaleza 2013) and the Ethics Committee of Clinical Research from the Government of Aragón (CEICA; Spain) approved the study protocol. REF: CEICA: PI09/00063. 

### 2.3. Participants

A total of eight young elite male road cyclists (CYC) from different cycling teams in Spain, and eight physically active controls (CON), recruited from secondary schools and colleges, agreed to participate in the study (Table 1). Cyclists and controls were excluded if they were aged over twenty-one, or if they were unhealthy, with any chronic disease, musculoskeletal condition, or bone fracture, if they regularly took medicines, or if they had other habits affecting bone development. One of the cyclists presented extreme values for most bone markers and was consequently deemed an outlier, and excluded. 

These young road cyclists were regular participants in regional competitions, and had trained under supervision for an average of 13.5 h per week (h/week) over a minimum of two and a maximum of seven years prior to the study. Control subjects were involved in recreational sports (rugby, tennis, handball, or football) for at least 2 h/week with occasional weekend matches, however, none of them cycled for more than one hour per week. 

Subjects were asked to complete a medical and physical activity questionnaire, and to provide additional information in respect to physical activity, past injuries, medicines taken and known diseases.

### 2.4. Anthropometric Measurements

While the subjects were barefoot and clad in light indoor clothing, their body weight (kg) and height (cm) were measured with an electronic weighing scale (Type SECA 861; precision 100 g, range 0 to 150 kg) and a stadiometer (Type Seca 225; precision 0.1 cm, range 70 to 200 cm). Body mass index (BMI) was calculated as weight (kg) divided by height squared (m^2^).

### 2.5. Blood Collection and Biochemical Analysis

Fasting blood samples (10 mL) were drawn at 8:00 a.m., after 10 h overnight, through an indwelling venous catheter placed in a forearm vein. Then, serum was separated and stored at −20 °C for later analysis. In order to avoid diurnal variations in plasma levels of total [25(OH)D], blood samples were obtained at the same time of the day [27].

### 2.6. Bone Turnover Markers

The concentrations of serum OC, PINP and β-CTx were determined by an electrogenerated chemiluminescence immunoassay using an Elecsys 2010 analyzer from Roche Diagnostics GmbH (Germany). The kits used were also purchased from Roche Diagnostics GmbH. The measuring range for serum osteocalcin was 0.50 μg/L to 300 μg/L (defined by the lower detection limit and the maximum of the calibration curve). Values below the detection limit were reported as <0.50 μg/L. Values above the measuring range were diluted with Elecsys Diluent Universal at a concentration below 60 μg/L. Osteocalcin presented coefficients of variation (CV) of 4.0% and 6.5% at 15.5 μg/L and 1.4% and 1.8% at 68.3 μg/L. The measurement range for total PINP in serum ran over from 5 μg/L to 1200 μg/L. Intra- and inter-assay CVs were 1.8% and 2.3% at 274 μg/L and 2.9% and 3.7% at 799 μg/L. Values below the detection limit were reported as <5 μg/L. Values above the measuring range of 1200 μg/L were diluted with Elecsys Diluent Universal at a recommended concentration of 1100 μg/L. Analytical sensitivity (the lower detection limit) was <5 μg/L. β-CTx had intra- and inter-assay CVs of 1.0% and 1.6% at 3.59 μg/L and 4.6% and 4.7% at 0.08 μg/L. The range for measurements was between 0.010 μg/L and 6.00 μg/L, the analytical sensitivity or lower detection limit was 0.01 μg/L, and the functional sensitivity was 0.07 μg/L.

### 2.7. Vitamin D Status

Plasma [25(OH)D] was analyzed by ELISA using a kit (Octeia^®^ 25-Hydroxy vitamin D) from Immuno Diagnostic Systems (Germany) and measured with a Sunrise™ Photometer by Tecan (Mannheim, Germany). The sensitivity of this method is 5 nmol/L 25(OH)D and the variation was under 6%. The CV for the method was below 1%. The complete methodology has been described elsewhere [15].

### 2.8. Statistics

The normality of the data distribution was evaluated with the Kolmogorov–Smirnov test. All variables presented normal distributions, except PINP. Thus, this was logarithmically transformed although the original data are also reported.

The characteristics of the subjects were described using averages and standard deviation (SD) values for continuous variables. Independent t-tests were performed to evaluate differences between groups for descriptive variables. To determine how bone turnover markers and vitamin D status differed between groups, analyses of covariance (ANCOVAs) were applied, and adjusted for age. ANCOVAs for repeated measures ×2 (time) were performed between pre- and post-evaluation to determine the effects of cycling on bone metabolic markers and vitamin D status.

The probability value for the significance level was fixed at 0.05. Data were analyzed using the SPSS 19.0 statistical program (SPSS Incorporated, Chicago, IL, USA).

## 3. Results

Table 1 summarizes the descriptive characteristics of participants of the sample as a whole (cyclists and controls), before and after evaluation. The results showed no differences in any of the variables. 

### 3.1. Bone Metabolism Markers and Vitamin D

**Figure 1:**
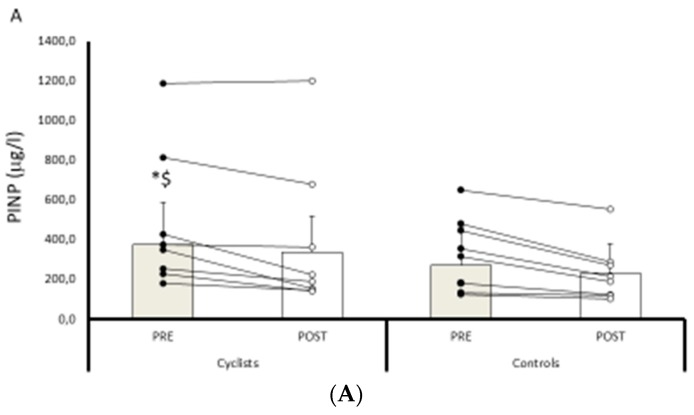

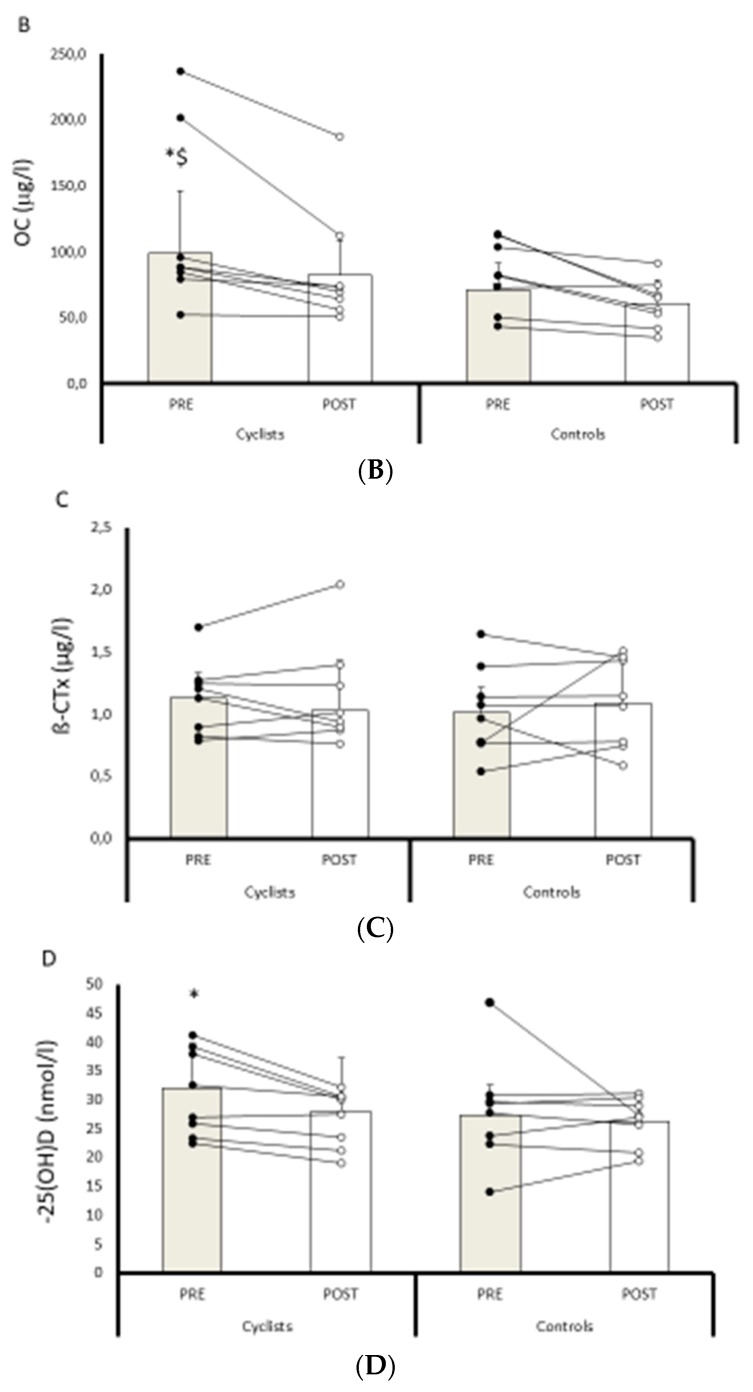
Panels (**A**–**D**) concentrations in cyclists (*n* = 8) and controls (*n* = 8), at pre- and post-evaluation. (**A**) Amino-terminal propeptide of type I precollagen (PINP). (**B**) Osteocalcin (OC). (**C**) beta-isomerized C-telopeptides (β-CTx). (**D**) Plasma 25 hydroxyvitamin D [25(OH)D]. Values are presented as the mean and SD. For final statistical analysis one cyclist has been removed (statistical outlier). The *p* values calculated with one-way analysis of covariance (ANCOVA), adjusting for age. * *p* < 0.05 compared to control group. ^$^ *p* < 0.05 compared to cyclists group.

Figure 1 shows the OC, PINP, β-CTx and the [25(OH)D] concentrations in cyclists (CYC) and controls (CON), before and after evaluation. No difference was observed between the groups for the markers or for 25(OH)D, either before or after evaluation (all *p* > 0.05). 

### 3.2. Changes within Group

Figure 1 shows intra-group results. With regard to bone metabolism, formation markers (OC and PINP) decreased in both the CYC and the CON groups (all *p* < 0.05). However, neither group showed changes in β-CTx (both *p* > 0.05). Nevertheless, [25(OH)D] decreased significantly in the CYC group (*p* < 0.01). 

### 3.3. Group-by-Time Interactions

No group-by-time interactions were found in any parameters. This suggests that both groups evolved similarly (all *p* > 0.05).

## 4. Discussion

The results of this one-year longitudinal study showed similar decreases in bone formation markers for both the CYC and CON groups over time, just as had been described in previous long-term studies relating to bone turnover markers in young female athletes [20,28,29]. Thus, the results suggest that this trend seems to be similar in athletes of both sexes, and in accordance with the results recorded by García-Marco et al. [30] in a study with an adolescent population (aged between 12 and 17), which detected a decrease in markers as puberty progressed in both boys and girls. As previously noted, bone remodeling decreases with age independently of whatever sport may be practiced, so growth may perhaps mask the effects of physical activity at these ages. The relationship between bone metabolism markers and bone mineral parameters in children and adolescents is not clear. Hence, further studies are needed to determine the role of bone turnover markers and physical activity on bone mineral acquisition during puberty.

No differences were found between them, when adult cyclists and runners were investigated in one piece of research [13], and other authors have determined normal ranges of bone markers in a group of young cyclists [31]. However, adult cyclists presented lower values for bone alkaline phosphatase (BAP) as compared to triathletes, swimmers, and controls, but yielded similar values for OC and β-CTX in another study [16].

Although there has been no previous investigation of changes in bone metabolism markers in adolescent cyclists, some researchers have analyzed BMD and BMC amongst this population, finding that cyclists had lower values for both variables than the controls did [10,25]. Moreover, Olmedillas et al. [10] reported greater differences in BMC and BMD between cyclists and controls in adolescents over 17 years of age. Since bone mass can be defined as the net product of bone formation and bone resorption [14], the present results for bone metabolic markers cannot provide support for the lower bone mass described previously [10]. This may be because bone metabolism markers are not site-specific and reflect bone remodeling of the whole skeleton [32]. It is not clear how metabolic markers reflect bone changes during growth and this topic requires further research.

Although we have not found significant differences in bone remodeling markers between cyclists and controls, other researchers show that cycling compromises the acquisition of peak bone mass throughout life [12,13,14,25,26]. One possible explanation for these differences might be the limited number of participants in the two groups in this study. Longitudinal investigations with adolescent cyclists would be needed to gain an understanding of the causes of this decrease in bone mass, and to act to prevent osteoporosis in these subjects in the future. A conceivable hypothesis would be that, even with the same metabolic activity, metabolic efficiency may be compromised because of physiological or nutritional interactions [33,34,35]. For instance, a major finding in this work was that 25(OH)D decreased significantly in the CYC group, but not in the CON group. Vitamin D deficiency is associated with decreased bone mass, because it compromises calcium absorption and impairs bone accumulation [21]. A direct association between 25(OH)D and BMC has been described previously [22,23,24]. In children, vitamin D deficiency is associated with demineralized bones and with rickets [22], so an adequate consumption of vitamin D is essential in preventing this disease. Low intakes of vitamin D, relative to the recommended dietary allowance (RDA) in Spanish children were observed by Gómez-Bruton et al. [36]. In particular, active adolescents did not consume the amount of vitamin D set for their age group, with cyclists being the group farthest away from meeting the recommendations. On the basis of their results, these low intakes are of concern in cyclists, because they showed lower values of BMC and BMD than controls. It is possible that an insufficient intake of vitamin D is among the principal causes of decreased bone mass in adolescent cyclists, as previously described by Olmedillas and colleagues [10]. Potential diurnal variation in plasma levels of total [25(OH)D], due mainly to variations in plasma volume previously described [27], have been taken into consideration. Therefore, in our study, blood extractions were collected at the same time in the morning, under the same conditions for all participants in pre- and post-evaluation.

The main limitation of our study is a relatively small number of subjects, however, the practice level of the participants should be taken into account. These cyclists were training for at least the last two years for a mean time of 13.5 h/week and participating in competitions during this time. An additional issue, is the range of age of the participants (mean, 17 years), as it is a challenge for their trainers to maintain the adherence of this level of training for long periods with a high level of commitment. Actually, we enrolled 25 cyclists in the first evaluation, however, due to most of them abandoning the training and to specific abandonments of the study, the final sample is the one that is represented in this study. The study’s principal strength is its longitudinal design (a one-year long-term design with two measurements in the season) and the specificity of the sample according to age and level of training. To date, there have been very few longitudinal studies that have analyzed the effects on biochemical markers in boys derived from practicing sport including cycling and gymnastics [37,38]. Further research, with dual energy X-ray absorptiometry (DXA) measurements and the assessment of a wider spectrum of bone osteoanabolic and catabolic parameters could provide a better understanding of the net process of bones.

## 5. Conclusions

The similar concentration of markers in cyclists and controls seems to indicate similar metabolic activity. As different levels of bone mass had previously been recorded in these cyclists, metabolic efficiency may have been compromised by other concurrent factors. Decreased vitamin D levels were, in fact, noted in the cyclists over this one-year period, which in the end could be detrimental to the future bone health of these individuals.

## Figures and Tables

**Table 1:** Descriptive characteristics of the sample. BMI, body mass index; SD, standard deviation.

	PRE						POST					
	Cyclists *n* = 7			Controls *n* = 8			Cyclists *n* = 7			Controls *n* = 8		
	Mean		SD	Mean		SD	Mean		SD	Mean		SD
Age (years)	16.3	±	0.9	15.8	±	1.5	17.6	±	1.2	16.9	±	1.5
Height (cm)	171.1	±	7.5	173.3	±	8	173.6	±	8	174.5	±	6.6
Weight (kg)	57	±	5.8	66.1	±	15.1	62.8	±	6.6	67.1	±	15.1
BMI (kg/m^2^)	19.5	±	1.7	22	±	4.1	20.9	±	1.8	22	±	4.8
Years of cycling training (years)	2.6	±	2.8				3.6	±	2.8			
Hours of cycling training (h/week)	10.5	±	7				13.5	±	5.2			

BMI: Body mass index; SD: Standard deviation.
